# Governing for Care Integration: Evidence from Spain’s Health and Social Care

**DOI:** 10.5334/ijic.11010

**Published:** 2026-10-22

**Authors:** Angel González de la Fuente, Arturo Álvarez-Rosete, Sara Calderón-Larrañaga, Joan Costa-Font

**Affiliations:** 1 Amsterdam UMC location University of Amsterdam, Department of Public and Occupational Health, Amsterdam, The Netherlands; 2 Amsterdam Public Health Research Institute, Quality of Care, University of Amsterdam, Amsterdam, The Netherlands; 3 International Foundation for Integrated Care (IFIC), Spain; 4 Nuffield Department of Primary Care Health Sciences, University of Oxford, Oxford, United Kingdom; 5 The George Institute for Global Health, School of Public Health, Imperial College London, London, United Kingdom; 6 Department of Health Policy, London School of Economics, London, United Kingdom

**Keywords:** integrated care, health and social care integration, health system and services research, multi-level governance, policy, Spain

## Abstract

**Background:**

Integrating health and social care is particularly challenging when responsibility is distributed across multiple levels of government and sectors, yet the governance conditions that enable or hinder integration remain poorly understood. Spain offers a valuable comparative setting: while its Autonomous Communities share a common national framework and hold responsibility for both health and social care, they have developed distinct governance models for integration.

**Objective and methods:**

We examine the multi-level governance of integrated health and social care drawing from evidence in Spain and using the TAPIC framework (Transparency, Accountability, Participation, Integrity and Policy Capacity) to identify barriers and enablers. This two-phase qualitative study covered six Autonomous Communities with established integrated care initiatives, combining six semi-structured interviews with senior regional leaders and three in-depth case studies based on document review and multi-stakeholder focus groups. Coding was deductive against TAPIC, attending throughout to how authority, resources and responsibilities are distributed across levels of government; a second, inductive stage, reported in the Methods, derived the CARE framework from the same dataset.

**Findings:**

We found substantial variation across all TAPIC dimensions, with governance weaknesses arising primarily from relationships between levels of government rather than within individual tiers. Fragmented information systems and unclear cross-sector responsibilities undermined transparency and accountability, while participation often remained procedural and failed to meaningfully engage underserved groups. Integrity challenges stemmed from blurred organisational boundaries and ethical dilemmas surrounding resource allocation and data sharing. Policy capacity varied widely, ranging from regions with strong digital infrastructure to those reliant on voluntary resources. Building on these findings, we developed the CARE framework—Collaboration, Adaptation, Resource Use and Empowerment—which translates TAPIC’s diagnostic insights into actionable governance functions.

**Conclusions:**

Multi-level governance gaps, particularly around transparency, accountability and consequential participation, continue to constrain integrated health and social care in Spain. CARE offers a practical governance framework to support implementation by strengthening collaboration, adaptation, resource use and empowerment across sectors and levels, though it is derived from a single national context and requires validation elsewhere.

## Introduction

As people live longer, more are living with complex, long-term conditions that require coordinated support from multiple services and professionals, yet this often results in fragmented care, duplication and inefficiency [1]. Integrated health and social care has emerged as a key policy response to the fragmentation of services associated with ageing populations and increasingly complex care needs. It involves the coordinated planning, delivery and governance of services centred on people’s needs across providers, sectors and care settings over time. The overarching aim is to ensure that individuals experience seamless, person-centred care while improving continuity, equity of access, service quality, patient and caregiver experience and the efficiency and sustainability of health and social care systems [2]. A recurring explanation for fragmentation is weak governance: the processes through which care is steered beyond formal government, what Rhodes described as ‘the new method by which society is governed’ [3]. The governance of integrated care encompasses ‘the total package of leadership, accountability and supervision in the local setting in an area or region’ [4] and is central to widely used frameworks including the International Foundation for Integrated Care’s (IFIC) Nine Pillars, SCIROCCO, the WHO people-centred services framework and SELFIE.

Empirical work on how governance shapes the functioning of integrated care nonetheless remains scant [5]. Coordination across providers, sectors and levels depends on collaboration, shared accountability and coherent decision-making, so understanding how governance enables or constrains it is essential for systems meeting the needs of ageing and clinically complex populations [6]. Primary care is widely recognised as a natural locus for integration, given its role in first contact, relational continuity and close engagement with patients, families and communities; strong primary care is associated with better coordination, more person-centred care and improved management of chronic conditions [7]. How far it acts as an integrative hub depends on its position within broader governance arrangements.

Multi-level governance is central to organising health and social care, particularly for managing complex systems and common-pool resources [8]. We use the term in a specific sense, distinct from governance understood as a set of organisational or procedural attributes: the distribution and negotiation of authority, decision rights, financing and responsibility between tiers of government—national, regional and local—and between public authorities and non-state actors [8]. Attributes such as transparency or accountability, by contrast, describe qualities of decision-making observable at any single tier. The two are entangled: accountability becomes diffuse precisely because responsibility is split across tiers, and participation becomes procedural when the tier convening it lacks the relevant decision rights. We therefore treat governance attributes as conditioned by inter-level relationships, asking not only whether an attribute is present, but where it is anchored, who holds the corresponding authority and how disagreements between levels are resolved.

Effective governance therefore depends on the quality and stability of relationships across levels. A core tenet of good governance is that actors assume roles aligned with their expertise, resources and institutional capacity to develop, adapt, oversee and enforce the rules governing provision [8,9]; where those roles are unclear or poorly coordinated, governance itself becomes a barrier to integration and system performance.

Spain offers a relevant setting given its decentralised health system, in which responsibilities are shared across national, regional and local levels [10]. Central government retains responsibility for overarching framework regulation, equity and coordination; the Autonomous Communities hold substantial autonomy in organising, financing and delivering healthcare [11,12], and local providers oversee implementation. Spain’s primary care–based model, with territorially organised multidisciplinary teams serving defined populations, provides a strong foundation for continuity. Although decentralisation can serve as an institutional design to further integration, it can also exacerbate fragmentation where health and social care responsibilities are split across departments, funding streams and institutional logics [13,14,15].

Integrated care models aim to improve efficiency, prevent avoidable hospitalisations and support delivery across residential and home settings [16]; in Spain these goals inform initiatives such as the Spanish Healthy Society 10-Year Strategy, which integrates primary care, specialist services, mental health and social support networks around the person [17]. Realising them requires governance capable of aligning actors, incentives and resources across sectors and levels, since structural challenges—fragmented systems, misaligned funding, unclear roles—interact with cultural barriers such as professional silos and differing values [2,18,19,20].

This paper examines the governance barriers and enablers influencing integrated health and social care in Spain across multiple levels of government, with particular attention to chronic conditions and the coordinating role of primary care. It contributes one of the first in-depth, multi-regional analyses of governance for integrated care in a highly decentralised system where responsibility for health and social care is institutionally divided. In doing so, it maps how integrated care and continuity of care are conceptualised, governed and implemented across Spain’s Autonomous Communities, highlighting the governance arrangements that facilitate or constrain integration [21,22], with lessons for policymakers, managers and researchers in devolved health systems.

## Methods

This study employed a two-phase exploratory qualitative design to investigate barriers and facilitators in the multi-level governance of integrated care in Spain, using the Transparency, Accountability, Participation, Integrity and Policy Capacity (TAPIC) framework [23]. TAPIC, developed by Greer et al. [24], sets out five dimensions of governance shaping decision-making and outcomes (Table 1). We applied it not as a checklist for a single tier but as a lens on relationships between tiers: for each dimension we asked where authority sits, how it is exercised across levels and where friction arises.

**Table 1:** TAPIC framework for analysing ‘good’ governance.Source: Adapted from Greer et al. [24].


DIMENSION	DEFINITION

**Transparency**	Institutions clearly communicate decisions and their rationales to the public and stakeholders.

**Accountability**	Individuals and institutions provide comprehensive accounts of their actions and face consequences when standards are not met.

**Participation**	Stakeholders are meaningfully involved in deliberations, with protections against reprisals.

**Integrity**	Well-defined processes exist for representation, recruitment and enforcement.

**Policy capacity**	Ability to design and implement resource-aligned, strategically focused policies.


The study focused on six Autonomous Communities—Catalonia, the Basque Country, the Community of Madrid, Castile and León, Asturias and Andalusia—purposively selected for their well-established integrated care programmes or sustained policy commitment to integrated care over the preceding decade. From the 73 examples of successful integrated care practice documented in Spain between 2014 and 2018 by the *Observatorio de Modelos Integrados en Salud* (OMIS; Observatory of Integrated Models in Health) [25,26], we selected six initiatives, one per region, and developed three as in-depth case studies (Castile and León, Asturias and Andalusia; Table 2), chosen to vary in governance form and in the balance between health, municipal and third-sector actors. For each we undertook a structured desk review of regional legislation, strategy documents, care pathways and evaluation reports to reconstruct the formal governance architecture and trace key policy decisions.

**Table 2:** Case study selection details.


PROGRAMME	STUDY SITE	DESCRIPTION	ROLE OF HEALTHCARE PROVIDERS	ROLE OF SOCIAL CARE/COMMUNITY ACTORS

**EFFICHRONIC Project**	Asturias	Funded under the Third EU Health Programme (grant 738127) and delivered June 2018–February 2020 across five countries, with Asturias as the Spanish site. Evaluated the effectiveness and cost-effectiveness of the Chronic Disease Self-Management Programme (CDSMP) for socially vulnerable adults with chronic conditions and their carers, through structured group workshops with standardised outcome and economic evaluation.	Regional health system actors (Health Service of the Principality of Asturias; Public Health Directorate) sit within the consortium; health professionals contribute to recruitment and referral and co-lead workshops alongside lay leaders.	Recruitment targets people missed by routine services, using community-based recruitment sites and vulnerability mapping.

**Sevilla Contigo, Ciudad Compasiva**	Andalusia	Compassionate city initiative launched in November 2015 as a demonstration of the ‘Todos Contigo®/All with You®’ methodology, supporting people with advanced chronic illness, end-of-life needs and bereavement. Combines community awareness and training, mapping of support networks and individualised interventions, operationalised through a community promoter and the RedCuida protocol.	The Andalusian Health Service, health centres and hospitals support and refer people with advanced illness; primary care and palliative care teams act as referrers and partners in the pathway.	Municipal and community actors (City Council, community social services, third-sector organisations, volunteers) activate the support network, led by community promoters who typically hold social work profiles.

**Integrated Care Process for Complex Pluripathological Chronic Patients**	Castile and León	Official regional integrated care process within the chronic care strategy, aiming to improve health and quality of life through integrated care and shared responsibility across levels, including social services. Uses population segmentation based on adjusted morbidity groups (Grupos de Morbilidad Ajustada, GMA) (risk groups G0–G3), positions primary care as the pillar of coordination and specifies interfaces with continuity of care units (unidades de continuidad asistencial, UCA) and health and social care convalescence units (unidades de convalecencia sociosanitaria, UCSS).	Primary care medicine and nursing lead identification, intake, assessment and care planning; coordination involves internal medicine and geriatric interconsultation and a case manager nurse linked to the UCA.	Social work in primary care contributes to integral assessment and care planning; hospital social work manages transitions, including transfer to UCSS. The pathway explicitly frames chronic care as requiring attention to the social and family environment.


In Phase 1 we conducted semi-structured interviews (SSIs) [27] with six key informants, one per Autonomous Community, all holding strategic responsibility for integrated health and social care at regional or sub-regional level (directors-general, senior programme leads or equivalent). The guide covered the origin and evolution of integrated care strategies, institutional arrangements for collaboration, decision-making and accountability structures, stakeholder participation and perceived barriers and facilitators at different governance levels, including the division of competences and resources across national, regional and local authorities.

In Phase 2 we conducted one focus group per case study (three in total, five participants each) to explore governance processes, day-to-day coordination and implementation from a multi-stakeholder perspective, structured by a TAPIC-based topic guide [24] adapted to each initiative (Appendix; Supplementary file 1). Participants were purposively sampled among stakeholders directly involved: primary care professionals, hospital clinicians where relevant, social workers and managers of social and community services.

Interviews (approximately 60 minutes, February–June 2025) and focus groups (90–120 minutes, June–December 2025) were conducted in Spanish via Zoom or Microsoft Teams, audio-recorded with consent, transcribed verbatim and analysed using a framework approach [28,29]. Within-case analyses triangulated interviews, focus groups and documents into narratives on governance arrangements, implementation trajectories and perceived outcomes; cross-case comparison identified patterns in the governance configurations shaping integrated care across the three regions. Reporting follows the Consolidated Criteria for Reporting Qualitative Research (COREQ) [30]. Quotations are identified by region (R1–R6), source (SSI = interview; FG = focus group) and participant number.

### Development of the CARE framework

CARE was not an incidental product of the TAPIC coding but the output of a distinct, sequenced analytic stage. Because the relationship between the two frameworks is central to our contribution, we set that sequence out in full.

*Step 1: deductive coding*. All transcripts and documents were coded against the five TAPIC dimensions, producing the diagnostic map, at and between levels, that underpins the comparative findings below.

*Step 2: inductive coding of unclassified material*. Coding also generated material participants treated as consequential but that fitted no single TAPIC dimension, typically because it described what actors *do* to make coordination work rather than a quality of their decisions. This material, together with segments coded to more than one dimension, was open-coded without reference to TAPIC.

*Step 3: grouping by governance function*. Codes were grouped by the function they described rather than the attribute they evidenced. Four clusters recurred across all three case studies and both data types: working across organisational boundaries; revising arrangements when they fail; mobilising the resources coordination consumes; and giving users, carers and frontline staff genuine influence. These became Collaboration, Adaptation, Resource Use and Empowerment.

*Step 4: mapping back to TAPIC*. Each cluster was mapped onto the TAPIC dimensions from which its evidence had originally been coded, making explicit that CARE is not independent of TAPIC: every function draws on two or more TAPIC attributes, reorganised around implementation rather than diagnosis.

*Step 5: validation within the research team*. The framework was tested by re-coding a subset of transcripts using CARE, confirming that each dimension was evidenced in all three case studies and discussing until consensus; provisional clusters not independently supported across sites were collapsed into the four retained dimensions.

CARE was therefore derived from, and so far tested only against, this Spanish dataset; we return to the implications in the Discussion.

### Involvement of people with lived experience

People with lived experience of chronic illness, disability or caring were not involved in formulating the research question, designing the study, conducting fieldwork, analysing data or interpreting findings: the study examined governance from the standpoint of those formally responsible for designing, financing and coordinating services, and participants were accordingly policymakers, managers, clinicians, social workers and community-sector professionals. Lived experience entered the dataset only indirectly, through professionals who routinely represent users and carers and through two case-study initiatives (EFFICHRONIC and Sevilla Contigo, Ciudad Compasiva) built on lay leadership and community co-production.

### Ethics approval

The study received ethical approval from the Research Ethics Committee at the London School of Economics (LSE), Ref: 375452. All participants received written information about the study and gave written informed consent; quotations are reported anonymously.

## Findings

Findings are organised by TAPIC dimension, reporting both the attribute itself and the inter-level dynamics conditioning it. We first summarise the formal governance architecture of the three case-study regions, then the comparative TAPIC findings and finally how cross-case patterns informed CARE.

### Regional governance architecture in the three case studies

To anchor the TAPIC results empirically, we reconstructed the formal governance baselines from the regional decrees and strategic documents in force when the selected initiatives entered the OMIS registry, then checked whether these remained operative during our 2025 fieldwork. Asturias organised coordination around a decree-based, three-tier structure separating strategic steering, technical support and territorially organised implementation, consolidated in the Health and Social Care Plan 2019–2021 [31]. In Andalusia, “Sevilla Contigo, Ciudad Compasiva” (launched 2015) predates later region-wide frameworks, so the case captures municipal and third-sector co-production alongside the architecture then in force: Decree 105/2019, creating a Directorate-General for Health and Social Care responsible for coordination planning and post-discharge follow-up [32]. In Castile and León, coordination rests on a long-standing regulatory framework operationalised through first-line Basic Coordination Teams, with the Fourth Health Plan (2016–2020) reorienting the health service towards integrated care and shared responsibility [33].

### Transparency

Transparency emerged as essential yet fragile. Stakeholders framed it first as clarity of purpose and process: accessible, regularly updated information on objectives, decision criteria and role expectations. Where this ‘governance transparency’ was routinely communicated rather than simply issued, participants described better alignment and fewer boundary misunderstandings.

*‘What we miss is not another plan; it’s a clear line of sight on priorities and decisions—what the objectives are, how choices are made, and what each actor is expected to do*.*’ [R3-FG-P4]*

Transparency was also understood as information openness: whether information exists, is accurate and trusted, is accessible beyond a narrow circle of experts and is comparable across services. Respondents highlighted gaps in eligibility criteria, referral routes and knowledge of available social and community resources, and selective sharing through informal networks. Digital systems were contingent enablers rather than transparency in themselves: advanced systems can deliver little transparency if information is incomplete or unusable, whereas modest ones suffice where disclosure is reliable.

These gaps were level-specific rather than uniform. Regional respondents reported reasonable visibility of their own strategic decisions but limited sight of how municipalities allocated social care resources; municipal and provider-level respondents reported the reverse, describing regional priority-setting as opaque and communicated late. Unable to observe the other’s rationale, each tier tended to read the other’s constraints as unwillingness, a misattribution that hardened over successive planning cycles.

### Accountability

Accountability arrangements varied substantially. The central issue was how far accountability is formalised across the health and social care interface: whether repeatable arrangements specify cross-sector responsibilities, establish monitoring routines, define escalation routes when coordination fails and enable corrective action. Formal intersectoral bodies were useful venues for role clarification and joint review, but accountability remained diffuse where responsibilities spanned departments, funding streams and organisational mandates.

*‘Accountability is a grey area. Everyone contributes to integrated care, but when things go wrong, it’s unclear who should step up. Co-governance agreements could help make roles and responsibilities more concrete*.*’ [R1-FG-P5]*

Documented health and social care planning and multi-level coordination bodies were perceived to offer a clearer ‘accountability map’ at strategic level, notably in Asturias and through Andalusia’s region-wide strategy. In Castile and León, routine coordination structures with explicit supervisory and reporting functions anchored responsibilities for recurring processes, though ambiguity persisted in complex cases spanning several services. Across regions, contractual and performance instruments remained output-oriented and rarely incorporated explicit integrated-care accountability, such as shared outcomes frameworks or cross-sector performance agreements, which reinforced responsibility-shifting at boundaries.

The underlying difficulty was less an absence of accountability than its asymmetry across levels. Health services answer upward to regional health authorities through contract-programme instruments; social services answer to municipal or provincial authorities under separate financing and reporting regimes. No respondent could identify a forum with authority to hold both chains to a shared outcome, so escalation stopped where the hierarchies diverged and cases falling between them were resolved, if at all, by personal negotiation. Relational governance—trust, consistent communication and capacity for conflict resolution—was the enabling condition for accountability to become genuinely shared.

### Participation

Participation was widely recognised as pivotal for legitimacy and problem-solving, yet respondents described substantial variation in who is involved, through which channels and whether participation connects to real decision points. Interviews and focus groups converged: participation often stops at consultation, with limited traceability from input to decision.

*‘Patients are invited to participate, but it’s often tokenistic. We need mechanisms where their input genuinely shapes policies, not just ticks a box for participation.’ [R4-SSI-P1]*

Where health and social care strategies existed, they created formal venues through which perspectives could in principle feed into cross-sector planning, though follow-through varied. Two systematic gaps were emphasised: inclusivity (marginalised groups and carers under-represented, frontline knowledge inconsistently incorporated) and consequentiality (limited evidence that participation affected priorities, pathways, resourcing or accountability). Against hierarchical cultures and rigid administrative routines, participants advocated institutionalised participatory structures paired with decision-tracking—what was proposed, what was decided and why.

Participation was also stratified by level. Formal consultative venues were concentrated at regional level, where strategy is written, while the decisions most visible to users and carers—eligibility thresholds, referral routes, the availability of home support—are taken at municipal or provider level, where structured participatory channels were sparsest. This mismatch between the level that consults and the level that decides helps explain why participation was so often experienced as procedural.

### Integrity

Integrity, understood as the coherence and ethical defensibility of governance, was described as a cornerstone of integrated care. Respondents framed it less as a matter of technical tools than of a shared normative framework: agreed principles clarifying what good integrated care looks like, how equity is protected and what can reasonably be expected of actors when resources are constrained. Where such principles were explicit, disputes about roles were fewer; where implicit or contested, integrity problems surfaced as inconsistent practice, boundary disputes and uneven decisions for similar needs.

*‘Clear role definitions have made a big difference in our region. The health and social portal allows us to align efforts, but when resources are tight, ethical tensions around prioritisation often emerge.’ [R3-FG-P2]*

Protocols and digital platforms supported integrity only insofar as they operationalised shared principles and legitimated cross-sector commitments. Proposed improvements centred on strengthening normative foundations—a charter of person-centred principles co-produced with stakeholders and service users—supported by joint training. Several ethical tensions were inter-level in origin: professionals applied regionally set prioritisation criteria to people whose social circumstances were assessed, and resourced, by a different administration operating under different eligibility rules.

### Policy capacity

Policy capacity was the most uneven TAPIC dimension but was consistently framed as governance capacity rather than resources alone: the ability to articulate a credible strategic vision, mobilise diverse actors around it, sustain alliances across sectors and translate intent into operational routines, monitoring and learning. Stakeholders linked it to population-oriented intelligence, deliberate workforce development and an interprofessional culture supporting boundary-spanning work.

*‘The ambition is there, but the tools and funding often fall short. Investing in staff training and infrastructure would make a huge difference.’ [R2-FG-P3]*

Workforce and digital infrastructure were secondary enablers, important insofar as they support governance capacity rather than constituting it. Capacity was unevenly distributed not only between regions but between tiers within them: regional authorities generally held the analytical and digital capability, while municipalities and providers, which carry most of the day-to-day integration work, more often lacked the planning capability, protected time and data access to use it. Community and third-sector assets were valued as partners, but respondents cautioned that policy capacity is shown partly by whether governance arrangements sustain these alliances rather than allowing voluntary effort to fill gaps statutory services are legally required to cover, which risks entrenching territorial inequity.

### From TAPIC findings to the CARE framework

TAPIC served as the primary analytic framework because it offers a structured, theory-informed lens for comparing how decisions are made, justified and implemented in complex systems, and it proved diagnostically powerful: applied to inter-level relationships across the six regions, it showed that fragility clusters at the junctions between tiers rather than within them. What it did not capture was the question participants kept returning to: not how good governance is, but *what governance work has to be done, by whom, for coordination to hold*. Frontline professionals described governance as enacted at organisational boundaries: collaborating, adapting when routines fail, mobilising resources, enabling voice from service users. This is a question of function rather than attribute, and it is the gap CARE fills.

CARE—Collaboration, Adaptation, Resource Use and Empowerment—was derived from the same dataset through the five-step process set out in the Methods. Each dimension reorganises evidence originally coded to two or more TAPIC attributes around the actions required to move from intent to implementation, underscoring that governance for integrated care is shaped not only from above but constructed from below, through routine coordination, negotiation and learning. Table 3 sets out each dimension with its definition and illustrative evidence, showing not whether a region ‘has’ CARE but where each function is institutionalised and where gaps persist between formal design and daily practice.

**Table 3:** How CARE dimensions are expressed in practice: definitions and verbatim examples.


CARE DIMENSION	SHORT DEFINITION	ILLUSTRATIVE EVIDENCE

**Collaboration**	Structured cross-sector working through shared objectives, joint responsibility and regularised relationships across health, social care and community actors	*‘Strong leadership is not just about coordination; it’s about building trust and creating shared goals across sectors. Without this, integration remains a distant goal.’ [R5-SSI-P1]* *‘What makes integration work is shared leadership—someone who can align health and social care around one set of priorities, not two parallel agendas.’ [R2-SSI-P1]* *‘When we sit at the same table regularly, things move. Without a routine space to meet, coordination depends on whoever has a good personal contact that week.’ [R1-FG-P3]*

**Adaptation**	Capacity to revise governance arrangements, roles and practices in response to changing needs, evidence and implementation learning	*‘Systems need to evolve as care needs do. A rigid framework doesn’t work for today’s patients or tomorrow’s challenges.’ [R1-FG-P1]* *‘We realised the pathway looked fine on paper but failed in practice, so we redesigned the steps with the team and tested a simpler version the following month.’ [R2-FG-P2]* *‘What helped was having a feedback loop—when the coordination process broke down, we reviewed the case, changed the protocol, and everyone knew the new rule.’ [R2-FG-P5]*

**Resource Use**	Sustainable alignment and allocation of statutory and enabling resources (workforce roles, time, financing and infrastructure) to support integrated delivery	*‘The biggest resource is time. Coordination is treated like an “extra”, so it happens after hours or not at all.’ [R3-FG-P4]* *‘We can’t deliver integrated care on goodwill. If the system wants joint working, it has to fund the roles and the back-office support that make it possible.’ [R3-FG-P2]* *‘Digital tools help, but they don’t fix under-resourcing. Without staffing and training, platforms become another task rather than an enabler.’ [R5-SSI-P1]*

**Empowerment**	Meaningful influence of patients, carers, communities and frontline professionals on priorities, decisions and accountability	*‘Empowerment isn’t just involvement; it’s influence—having a real say in policies and decisions that directly impact us. We’re invited to consultations, but the decision is already taken. Empowerment would mean seeing what changed because of what we said.’ [R1-FG-P1]* *‘If you want meaningful participation, you need decision-tracking: what was proposed, what was accepted, what was rejected, and why.’ [R3-SSI-P1]* *‘Frontline staff and service users know where integration fails, but their input isn’t treated as expertise unless there’s a mechanism that turns it into action.’ [R6-SSI-P1]*


## Discussion

This paper examined the governance of integrated health and social care in a decentralised system, demonstrating that governance challenges extend beyond organisational arrangements to the relationships between actors and levels of government. Across the three case-study regions, the TAPIC analysis identified recurring vulnerabilities, including limited transparency, diffuse accountability, uneven participation, integrity challenges at organisational boundaries and substantial variation in policy capacity. The cases reach these vulnerabilities by different institutional routes—a decree-based structure with a dedicated plan (Asturias); a strategy-led model with intersectoral steering commissions (Andalusia); routinised coordination through case-management teams supported by area-level commissions (Castile and León)—and that three such different architectures produce a similar profile of weakness is itself informative: the constraint appears to lie in how authority and resources are divided between levels, not in the structure chosen at any one of them.

These arrangements delivered effective governance only when routinely activated: through visible cross-sector leadership, clear role expectations, escalation routes when coordination fails, communication sustained beyond informal networks and institutionalised feedback rather than reliance on individual champions. Primary care was the practical backbone of this work: family doctors and community nurses acted as the day-to-day ‘connective tissue’ translating formal agreements into coordination across hospitals, social services and community actors, and coordination was more reliable where governance explicitly enabled them through protected time, information-sharing and clear escalation routes.

The three regions show uneven but visible policy maturation. By 2026 Andalusia had moved beyond the organisational mandates of 2019 to a region-wide strategy [34], supported by coordination commissions that set priorities and establish cooperation channels between health and social services; Castile and León has updated its chronic care strategy [35], reiterating integration with the social system and strengthening primary care’s coordinating role; Asturias still references the 2019–2021 plan, suggesting continuity in its decree-based architecture but also the need for a refreshed roadmap. The trajectory runs from structures to strategies; the road ahead lies in turning plans into routinised practice.

Our findings align with international research. Exley et al. [36] identify common European challenges: fragmented national policies, blurred accountability, insufficient attention to local realities and the structural dominance of acute over social care. Exworthy, Powell and Glasby [37] report persistent governance problems in England despite successive initiatives. Consistent with both, we observed enduring gaps between ambition and implementation, with local actors reconciling inconsistent incentives, fragmented information systems and overlapping accountability demands. Institutional context shapes how these tensions play out: in highly decentralised systems services follow parallel paths, leaving local teams to assemble responses without an overarching national framework, whereas in England overlapping national programmes and policy churn drive fragmentation [37]. These challenges appear systemic rather than country-specific, evident across centralised and decentralised systems alike [36,37]. Improving governance therefore requires more than organisational restructuring: sustained investment in governance capability, particularly in primary and community care; mechanisms that align accountability across organisations and levels; and institutionalised participation that empowers patients, carers, professionals and communities.

Governance does not operate in isolation from the other conditions shaping implementation, and our data point to four interactions. First, organisational incentives: where contract-programme instruments reward activity within a single sector, they implicitly penalise time spent coordinating across the boundary, so coordination survives on discretionary effort and is surrendered first under pressure. Second, resource allocation: separate budget lines, set by different administrations on different cycles, mean savings from integration accrue to one budget while costs fall on the other. Third, political commitment: respondents linked the durability of coordination structures to whether senior sponsorship survived electoral turnover. Fourth, sustainability: several initiatives, EFFICHRONIC among them, began as time-limited projects, and the transition to recurrent funding was where arrangements were genuinely tested. These are not external to governance but the material conditions determining whether arrangements are activated or remain formal.

Governance designed for single organisations does not match what collaborative arrangements require [38], and collaborative leadership emerged as a cornerstone of effective governance. Settings where leaders aligned priorities across sectors, built trust and brokered compromises were more likely to sustain integration [39,40,41,42,43]; fragmented leadership exposed misaligned goals and unresolved power imbalances. Leadership development focused on relational governance and conflict resolution is essential [44] but must be coupled with structural supports such as shared governance forums and clear co-governance mandates.

Adaptation is equally central. Modular care models, reconfigurable teams and timely data use enhance responsiveness, whereas rigid structures constrain it [45,46]; adaptable governance should permit adjustment without destabilising core systems [47]. It largely occurs at meso and micro levels, where frontline professionals modify referral criteria and roles. Such local workarounds become a deliberate learning strategy only if captured and institutionalised.

Empowerment emerged as a distinct governance dimension. Advisory boards, co-design structures and feedback loops can reduce tokenism, but become procedural where they lack decision rights, are poorly resourced or fail to show how input changes policy [48,49]. Empowerment cannot therefore be achieved through formal architecture alone: it requires cultural shifts recognising frontline knowledge and lived experience as legitimate expertise, alongside protected time, facilitation and training. In contrast to evidence emphasising participatory structures [36], our data foreground community-level empowerment: civil society networks acted not only as consultees but as co-producers able to reframe priorities and exert social accountability.

Resource disparities strongly influence governance outcomes. Consistent with research on equity and decentralisation [50,51,52,53], regions with greater fiscal and digital capacity coordinated services more effectively, while under-resourced areas faced fragmentation. This argues for incentive-based financial models that encourage resource sharing without undermining local autonomy. Extending beyond pooled budgets, our study shows how community assets, voluntary organisations and municipal resources sustain integration, raising concerns about dependence on precarious labour; CARE’s Resource Use dimension addresses the distribution, visibility and sustainability of non-health-sector resources often overlooked in integration finance debates [36].

CARE warrants more critical appraisal than newly proposed frameworks usually receive. Its dimensions are not independent of TAPIC, and we do not claim otherwise: each reorganises evidence drawn from two or more TAPIC attributes. The overlap is deliberate and the claim correspondingly modest: TAPIC asks what quality of governance a system exhibits, CARE what governance work must be done, and by whom, for coordination to hold. The reorganisation earns its place because the two questions have different addressees—TAPIC those who oversee systems, CARE those who run them—and because knowing that accountability is diffuse does not tell a manager what to do about it. Two limits remain. Derived inductively from a single national dataset, CARE may reflect features of the Spanish system—regional health services, municipal social services, a strong primary care base—rather than universal functions; and it has not been tested prospectively: we show that it organises our data coherently, not that it improves coordination. Validation should therefore examine its applicability across territorial and fiscal contexts, and whether stronger CARE performance is associated with more reliable coordination and better outcomes.

### Strengths and limitations

The study’s strengths lie in its comparative, multi-region design and its use of multiple qualitative sources—document review, senior-level interviews and multi-stakeholder focus groups—enabling triangulation between governance arrangements on paper and experiences of implementation, with TAPIC providing a shared lens for consistent cross-case comparison and the case studies adding contextual granularity. Four limitations qualify these findings. First, the study draws on a bounded set of regions, and the case studies were conducted in relatively advanced, pre-identified successful settings, which may under-represent barriers in lower-maturity contexts. Second, it includes no direct patient, carer or public perspectives, limiting conclusions about empowerment. Third, findings rely on self-reported accounts and documents and may be affected by social desirability, recall and positionality, with no linked quantitative indicators for corroboration against outcomes [54,55,56]. Finally, governance is dynamic, so the findings are a time-bound snapshot [57].

### Implications for policy and practice

Integrated care governance is strengthened less by creating new structures than by improving how organisations work at the health and social care interface. Using CARE as a practical guide, policymakers can institutionalise Collaboration through routine joint decision forums with named leads and escalation routes; enable Adaptation through feedback loops linking frontline learning to system steering; improve Resource Use by aligning priorities, incentives and interoperable information systems; and advance Empowerment by embedding meaningful participation, especially of underserved groups, in planning and oversight. We intend to develop these into policy briefs.

Future research should co-develop locally grounded governance arrangements that begin from integrative needs and specify the mechanisms enabling shared decision-making, trust and mutual accountability [58]. Longitudinal, mixed-methods studies are needed to assess how governance reforms influence outcomes, costs and sustainability, and how interoperable information systems and a parsimonious indicator set support learning [5,59]. Comparative work across decentralised systems could clarify which features transfer, and under what conditions.

## Conclusion

We examined how integrated health and social care governance operates within Spain’s decentralised system through interviews with regional leaders and three in-depth case studies. Our findings suggest that progress towards integration depends not only on formal governance arrangements—such as strategic plans, policy frameworks and intersectoral coordinating bodies—but also on how these arrangements function at organisational and governmental boundaries. Effective integration requires information systems that are interoperable and actionable, clearly defined and enforceable cross-sector responsibilities, participation that meaningfully influences decision-making and institutional capacity to support continuous learning and adaptation. This helps explain why integration remains uneven even where promising initiatives exist. We therefore offer CARE as a complement to TAPIC: where TAPIC locates vulnerabilities, CARE consolidates the work of addressing them, supporting a diagnose-and-act approach, though CARE requires validation beyond Spain. The wider lesson is that *formal structures deliver only when matched by routinely activated leadership, clear escalation routes, boundary-spanning roles and institutionalised feedback*.

## Additional File

The additional file for this article can be found as follows:

10.5334/ijic.11010.s1 Supplementary File. Appendix.
