# Supplementary material for: Governing for Care Integration: Evidence from Spain’s Health and Social Care
Source: Int J Integr Care. 2026 Oct 22;26(4):3. doi: 10.5334/ijic.11010 (PMC13693581; doi:10.5334/ijic.11010)
Supplement: Supplementary file 1 [file ijic-26-4-11010-s1.pdf]

Governing for Care Integration: Evidence from Spain's Health and Social Care.

Appendix

Topic Guide for: Semi-structured interviews (senior/key stakeholders)

Purpose

To elicit senior stakeholders' perspectives on how integrated care governance operates in practice within decentralised settings; identify governance bottlenecks and enabling conditions; and inform selection/focus of case-study regions.

Opening

- Brief introduction, consent, confidentiality, permission to record.
- Role and responsibilities: "Could you briefly describe your role and how it relates to integrated care and/or socio-health co-ordination?"
- Context: "What integrated care priorities or initiatives are most salient in your region at present?"

Transparency

- "How are strategic priorities and decisions about integrated care communicated across organisations and sectors?"
- "What information is routinely shared (or not shared), with whom, and through which channels?"

Accountability

- "How are responsibilities for integrated care defined across health, social care and other partners?"
- "What happens when co-ordination fails or goals are not met—who is expected to act, and through which escalation routes?"

Participation

- "Which stakeholders are involved in shaping integrated care priorities (patients, carers, professionals, municipalities, third sector)?"
- "How is stakeholder input linked to decision-making (e.g., traceability from proposals to decisions)?"

Integrity

- "What shared principles or standards guide integrated care decisions (e.g., person-centredness, equity, privacy)?"
- "How are ethical trade-offs managed when resources are constrained?"

Policy capacity

- "What capabilities are most critical to sustain integrated care over time (leadership, alliances, learning systems, workforce skills, population intelligence)?"
- "Where are the main capacity gaps between strategic ambition and implementation?"

Conclusion

- "If you could change one governance feature tomorrow to strengthen integrated care, what would it be and why?"

Topic Guide for: Focus groups (case-study regions)

Purpose

To capture operational experiences of integrated care governance at boundaries (health–social care–community), validate/contrast senior perspectives, and identify concrete governance practices and bottlenecks.

Format

- 60–90 minutes; 4–6 participants; mixed roles where feasible (health, social care, municipal/community/third sector).
- Ground rules: confidentiality, respectful turn-taking, no naming individuals/organisations in examples.

Transparency

- "How are decisions about integrated care communicated to stakeholders in practice?"
- "What challenges exist in keeping processes transparent?"

Accountability

- "Who is responsible for the success of integrated care programmes in practice?"
- "What mechanisms ensure accountability when goals are not met?"

Participation

- "How are different stakeholders, including patients, involved in decision-making?"
- "What barriers limit participation?"

Integrity

- "Are roles and responsibilities clearly defined across organisations?"
- "How are ethical standards upheld in decision-making processes?"

Policy capacity

- "Does your organisation have the capability to develop and implement integrated care policies effectively?"
- "What gaps exist between policy goals and the practical capacity to deliver?"

Closing

- "What are the top two governance changes that would make integrated care work better here?"
- Summarise key points back to the group for confirmation ("member check").
